# Two years of operation of the COMPARTE care programme for complex chronically ill patients/Dos años de funcionamiento del Programa para la Atención de Pacientes Crónicos Complejos COMPARTE

**Published:** 2012-05-29

**Authors:** Ignacio Vallejo Maroto, Antonio Fernández Moyano, Jose María Páez Pinto, Eva Martín Sánchez, Francisco Alemany Lasheras, Sergio González Limones, María Victoria Ruiz Romero

**Affiliations:** San Juan de Dios del Aljarafe Hospital, Bormujos, Sevilla, Spain; San Juan de Dios del Aljarafe Hospital, Bormujos, Sevilla, Spain; Aljarafe Health Region, Sevilla, Spain; Aljarafe Health Region, Sevilla, Spain; San Juan de Dios del Aljarafe Hospital, Bormujos, Sevilla, Spain; Aljarafe Health Region, Sevilla, Spain; San Juan de Dios del Aljarafe Hospital, Bormujos, Sevilla, Spain

**Keywords:** chronic diseases, continuity of care, quality of life, enfermedades crónicas, continuidad asistencial, calidad de vida

## Introduction

Chronic diseases (CDs) are a priority health problem. These are conditions that healthcare is not able to cure, and for patients they mean a progressive deterioration of their health and loss of independence. Their prolonged and unpredictable course leads to a loss in quality of life and high use of healthcare and social resources. Since April 2009, our hospital (San Juan de Dios de Aljarafe Hospital) and its catchment area (Aljarafe Health region) have operated a specific programme to provide integrated and longitudinal care.

### Description of the intervention

The objective of our programme is to promote home monitoring of CDs and improve patient quality of life. The programme aims to achieve the aforementioned objectives through the development of three areas: scientific and technical services; social and family care; and the coordination of care and safety. Across these areas, there are six main lines of development: stratification of patient risk; comprehensive assessment of patients (clinical, functional, psychological and social and family); provision of pharmacological treatment and care based on the best available evidence; self-care education; sustained coordination of care; and development of professional skills. These lines have been translated to 11 operational interventions: patient identification; integrated assessment and care; standardisation of procedures; rational use of medicines; self-care education; sectorisation of the inpatient and outpatient areas; transfer on discharge; linkage to electronic health records; follow-up in primary care; close observation to detect exacerbations; and development of professional skills. Currently, the programme is applied in the case of patients with heart failure, chronic obstructive pulmonary disease, multiple diseases, or cancer requiring palliative care. A multidisciplinary group of health professionals (general practitioners, and specialists, nurses from both levels of health care, social workers, IT specialists, etc.) participated in the establishment and implementation of the programme.

## Results

A total of 1308 patients have participated in the programme so far. Of these, 36.4% were included because they had multiple conditions, 30.8% for being readmitted, 22.4% for being classed as frail (following the criteria of the Department of Health of the regional Government of Andalusia [1]), 7.7% for having chronic respiratory disease, 8% for having heart failure and 2.7% because they required palliative cancer care. The average hospital stay that preceded inclusion on the programme was 9.04 days in 2009 and 10.37 days in 2010 (p=0.018). Most patients received a standard care plan during their hospital stay. Further, the main caregiver was given training in 40.5% of cases, while 8.1% of patients received direct training. The mortality rates in the hospitalisation episode were 18.6% in 2009 and 16.7% in 2010 (p=0.378). With regards to readmissions, 20.4% of participants were readmitted in 2009, while this rate fell to 10.7% in 2010. The inclusion criteria were similar for patients who were readmitted and the other participants. The mortality per episode was higher (35.89%). Once the programme was introduced, the average number of admissions was 1.5 among patients who were readmitted.

We highlight the falls in the demand for emergency services in primary care and in the number of GP consultations, by 6.5% and 7.2%, respectively, in 2010 compared to the previous year. The results of the evaluation of the activity in primary care are: 70% of patients were visited at home by their doctor and/or nurse and 33% received a joint visit within the agreed time frame (first 48–72 working hours).

## Conclusions

The programme aims to provide a multidisciplinary approach and integrated healthcare for patients with CDs. It has made it possible to reorganise and rationalise our resources. In addition, it promotes continuity between the different levels of care as well as patient safety. Patients and caregivers have become more involved in their own disease and care. The lessons learnt are that it is worth striving to promote team work as it can improve sometimes complex care by sharing the provision of care and it allows the transfer of this innovative experience to other areas of action, producing favourable health outcomes.

## Conference abstract Spanish

## Introducción

Las enfermedades crónicas (EC) constituyen un problema prioritario de salud. Se trata de procesos que los sistemas sanitarios no curan. En el paciente condicionan un progresivo deterioro y una pérdida de autonomía. Su curso prolongado e impredecible condicionará una merma en la calidad de vida y un elevado consumo de recursos sanitarios y sociales. Desde abril de 2009 nuestro centro hospitalario (Hospital San Juan de Dios del Aljarafe) y su área sanitaria de referencia (Distrito Sanitario Aljarafe) cuentan con un programa de atención específico para dar una respuesta integral y longitudinal a esta problemática.

## Descripción de la intervención

El objetivo de nuestro programa es mejorar el control en domicilio de las EC y potenciar la calidad de vida del paciente. Nuestro programa atiende las necesidades referidas, a través del desarrollo de tres áreas: científico-técnica; socio-familiar y cuidados; y el área de coordinación asistencial y seguridad. Estas áreas, se distribuyen en 6 líneas de desarrollo: estratificación del riesgo de los pacientes, evaluación integral (clínica, funcional, psico-afectiva y socio-familiar), tratamiento farmacológico y cuidados en base a la mejor evidencia disponible, educación en autocuidados, mantenimiento de coordinación asistencial, y desarrollo de competencias profesionales. Estas líneas asistenciales se han expresado finalmente en 11 intervenciones operativas: identificación de pacientes, evaluación y atención integral, homogeneización de procedimientos, uso racional de medicamentos, educación en autocuidados, sectorización de la planta de hospitalización y consultas, transferencia al alta, vinculación de historia clínica digital, seguimiento en atención primaria, atención a las reagudizaciones, y desarrollo de competencias profesionales. Se actúa sobre las siguientes entidades clínicas: insuficiencia cardíaca, enfermedad pulmonar obstructiva crónica, pacientes con pluripatología y paliativos oncológicos. Han participado en su puesta en marcha y desarrollo un grupo multidisciplinar de profesionales sanitarios (médicos de Atención Primaria y Atención Especializada, enfermeras de ambos niveles asistenciales, trabajadores sociales, informáticos, etc).

## Resultados

Se han incluido 1308 pacientes. Un 36,4% con criterios de pluripatología, un 30,8% por reingreso, un 22,4% por fragilidad (criterios de la Consejería de Salud de la Junta de Andalucía [1]), un 7,7% por enfermedad respiratoria crónica, un 8% por insuficiencia cardíaca y un 2,7% por enfermedad oncológica paliativa. La estancia media del episodio de hospitalización que motivó la inclusión en el programa fue de 9,04 días durante el 2009 y de 10,37 días durante el 2010 (p=0,018). La mayor parte de los pacientes recibieron un plan de cuidados estándar durante su hospitalización. Se realizó un adiestramiento del cuidador principal en un 40,5% y directamente sobre el paciente en un 8,1%. La mortalidad en el episodio de hospitalización fue de un 18,6% en el 2009 y un 16,7% en el 2010 (p=0,378). Analizando el reingreso, en el año 2009 reingresaron un 20,4% de los incluidos en el programa; en el 2010 un 10,7% de los incluidos. Los criterios de inclusión en los pacientes que reingresan son similares a los del resto. La mortalidad intraepisodio es superior (35.89%). Los que reingresan generan una media de hospitalizaciones de 1,5 desde la puesta en marcha del programa. Resaltamos la reducción en la demanda en atención primaria de los servicios de urgencia en el año 2010 en un 6,5% con respecto al año anterior y un 7,2% en consultas de Medicina de Familia. Recogemos la evaluación de la actividad en Atención Primaria: el 70% recibieron una visita domiciliaria por su médico y/o por su enfermera y el 33% recibieron una visita conjunta en el plazo acordado (primeras 48–72 horas hábiles).

## Conclusiones

El programa apuesta por un abordaje multidisciplinar y una Atención Integral del paciente con EC. Ha permitido reorganizar nuestros recursos y racionalizarlos. Potencia las líneas de continuidad asistencial interniveles y las estrategias de seguridad. Hace partícipe al paciente y a su cuidador de su enfermedad y cuidados. La lección aprendida es una apuesta por el trabajo en equipo que trata de transformar una atención en ocasiones compleja, por una compartida y permite trasladar esta práctica innovadora a otros ámbitos de actuación con resultados en salud favorables.
